# BNT162b2 booster after heterologous prime-boost vaccination induces potent neutralizing antibodies and T cell reactivity against SARS-CoV-2 Omicron BA.1 in young adults

**DOI:** 10.3389/fimmu.2022.882918

**Published:** 2022-07-25

**Authors:** Alina Seidel, Michelle Zanoni, Rüdiger Groß, Daniela Krnavek, Sümeyye Erdemci-Evin, Pascal von Maltitz, Dan P. J. Albers, Carina Conzelmann, Sichen Liu, Tatjana Weil, Benjamin Mayer, Markus Hoffmann, Stefan Pöhlmann, Alexandra Beil, Joris Kroschel, Frank Kirchhoff, Jan Münch, Janis A. Müller

**Affiliations:** ^1^ Institute of Molecular Virology, Ulm University Medical Center, Ulm, Germany; ^2^ Institute for Epidemiology and Medical Biometry, Ulm University, Ulm, Germany; ^3^ Infection Biology Unit, German Primate Center – Leibniz Institute for Primate Research, Göttingen, Germany; ^4^ Faculty of Biology and Psychology, Georg-August-University Göttingen, Göttingen, Germany; ^5^ Central Department for Clinical Chemistry, University Hospital Ulm, Ulm, Germany; ^6^ Core Facility Functional Peptidomics, Ulm University Medical Center, Ulm, Germany; ^7^ Institute of Virology, Philipps University of Marburg, Marburg, Germany

**Keywords:** COVID-19, delta, B.1.1.529.1, BA.1, humoral immunity, memory T cells, ChadOx1 nCoV-19, vaccination interval

## Abstract

In light of the decreasing immune protection against symptomatic SARS-CoV-2 infection after initial vaccinations and the now dominant immune-evasive Omicron variants, ‘booster’ vaccinations are regularly performed to restore immune responses. Many individuals have received a primary heterologous prime-boost vaccination with long intervals between vaccinations, but the resulting long-term immunity and the effects of a subsequent ‘booster’, particularly against Omicron BA.1, have not been defined. We followed a cohort of 23 young adults, who received a primary heterologous ChAdOx1 nCoV-19 BNT162b2 prime-boost vaccination, over a 7-month period and analysed how they responded to a BNT162b2 ‘booster’. We show that already after the primary heterologous vaccination, neutralization titers against Omicron BA.1 are recognizable but that humoral and cellular immunity wanes over the course of half a year. Residual responsive memory T cells recognized spike epitopes of the early SARS-CoV-2 B.1 strain as well as the Delta and BA.1 variants of concern (VOCs). However, the remaining antibody titers hardly neutralized these VOCs. The ‘booster’ vaccination was well tolerated and elicited both high antibody titers and increased memory T cell responses against SARS-CoV-2 including BA.1. Strikingly, in this young heterologously vaccinated cohort the neutralizing activity after the ‘booster’ was almost as potent against BA.1 as against the early B.1 strain. Our results suggest that a ‘booster’ after heterologous vaccination results in effective immune maturation and potent protection against the Omicron BA.1 variant in young adults.

## Introduction

Vaccination against the severe acute respiratory syndrome coronavirus 2 (SARS-CoV-2) is the key strategy to control the coronavirus disease 2019 (COVID-19) pandemic (1) and has already reduced incidences, hospitalizations, and deaths in several countries (2). Unfortunately, waning humoral immunity over time (3) and the emergence of immune evasive SARS-CoV-2 variants of concern (VOC) (4) impair vaccine effectiveness (5) and allow rebounds in infection rates (6, 7). The winter of 2021/2022 and the following summer came with the challenge of decreasing population immunity as initial vaccinations date back to early 2021 and the sudden appearance and rapid spread of the highly mutated immune evasive Omicron VOC (PANGO lineages B.1.1.529; BA.1, BA.2 and BA.3, BA.4, BA.5) (8–16). Therefore, ‘booster’ vaccinations are of enormous relevance to reestablish efficient protection (17, 18) and have been shown to induce humoral and cellular immune responses also against the Omicron VOC (9–14, 16, 19–21). ‘Boosters’ are performed as additional single vaccinations with a vaccine not necessarily matching the previous regimen. Generally, boosting triggers humoral and cellular responses. However, the degree might vary dependent on the specific combination of the initial vaccination regimen and the ‘booster’ vaccine (22). In at least 11 states of the European Union, individuals have received an initially unscheduled heterologous primary vaccination regimen consisting of a ChAdOx1 nCoV-19 (Vaxzevria, AstraZeneca) prime followed by a BNT162b2 (Comirnaty, BioNTech/Pfizer) boost after 8–12 weeks (23). This schedule had not been evaluated in clinical trials before application, but proven effective (24). The immunological responses were even superior to homologous vaccinations (25–28). However, the effect of a ‘booster’ following this regimen has not yet been described.

Here, we closely monitored the antibody titers and memory T cell immunity in a heterologously vaccinated cohort of young adults (25) over 7 months of follow-up and assessed the effect of a BNT162b2 ‘booster’. Our data show that immunity gradually declines over the course of 5.5 months but antibody and memory T cell responses are restored and increased after the ‘booster’. Responsive T cells recognized all SARS-CoV-2 variants, while the Omicron BA.1 VOC efficiently evaded neutralization by antibodies induced by initial vaccination. Strikingly, in this young cohort of heterologously vaccinated individuals where the primary vaccination had a longer interval than typically in homologous vaccinations, the ‘booster’ induced humoral immune responses that neutralized the Omicron BA.1 VOC almost as effectively as the early B.1 strain.

## Materials and methods

### Study design

Our cohort of 26 hospital employees who received a primary vaccination consisting of a ChAdOx1 nCoV-19 prime followed by a BNT162b2 boost after an 8-week interval has been previously described (25) (**Table 1**). Of these individuals, 23 agreed to participate in a follow-up study determining the course of immunity over time. Participants were eligible for recruitment if they had received a primary ChAdOx1 nCoV-19 BNT162b2 prime-boost vaccination. SARS-CoV-2 infections were determined by medical history and by measuring anti-SARS-CoV-2-nucleocapsid antibody levels before beginning and at the last time point of the study. One convalescent individual was detected and excluded from all statistical analyses. At 6.5 months after the primary vaccination, 18 participants decided to get a BNT162b2 ‘booster’ vaccination. Serum samples were taken every 1.5–2.5 months. In addition, of those participants who received a ‘booster’, 12 agreed to donate peripheral blood mononuclear cells (PBMCs).

**Table 1:** Study participants:.

	Serum			T cells		
	Total	m	f	Total	m	f
	**Longitudinal follow-up**					
**Participants**	23	8	15	12	6	6
**Age median**	29.5 (26-60)	32 (26-49)	30 (26-60)	36 (26-49)	36 (26-49)	35.5 (26-40)
**Prior SARS-CoV-2 infection**	1	0	1	0	0	0
	**‘Booster’**					
**Participants**	18	8	10	12	6	6
**Age median**	29.5 (26-49)	32 (26-49)	29.5 (26-40)	36 (26-49)	36 (26-49)	35.5 (26-40)
**Prior SARS-CoV-2 infection**	0	0	0	0	0	0

### Vaccine reactogenicity

Solicited adverse reactions (SAR) were self-reported by the participants *via* questionnaire following the ‘booster’ vaccination. Participants were asked to list symptoms, their duration (<1 h, few hours, 1 day or more than 1 day), and severity (mild (grade 1), moderate (grade 2), severe (grade 3)). Grading criteria were adapted from the US Department of Health and Human Services CTCEA (Common Terminology Criteria for Adverse Events, v4.03) (29), with grades 1–2 being considered for some symptoms, grade 1–3 for most, as previously described (25).

### Collection of serum and PBMC samples

At 5.5 months after the heterologous primary vaccination, and 2 weeks (antibody titers peaked around 14–19 days post initial heterologous vaccination (25)) after the BNT162b2 ‘booster’ (7 months post primary vaccination), blood was drawn into S-Monovette^®^ Serum Gel (Sarstedt) or S-Monovette^®^ K3 EDTA tubes. Serum gel collection tubes were centrifuged at 1,500 × g at 20°C for 15 min, aliquoted, and stored at -20°C until further use. PBMCs were obtained from EDTA tubes using density gradient centrifugation by Pancoll human (Pan Biotech, Germany), and erythrocytes were removed by ACK lysis buffer (Lonza, Walkersville, MD, USA). Mononuclear cells were counted for viability using a Countess II Automated Cell Counter (Thermo Fisher) with trypan blue stain and were cryopreserved in aliquots of up to 1 × 10^7^ cells in 10% DMSO in heat-inactivated FCS.

### Determination of antibody titers

IgG and IgM titers were measured as units per ml (U/ml) which correlates 1:1 with the WHO standard unit for the SARS-CoV-2 binding antibody units per ml (BAU/ml). To this end, serum was analysed using the commercial electrochemiluminescence Elecsys Anti-SARS-CoV-2 S immunoassay (Roche, Mannheim, Germany) by a cobas^®^ e801 immunoassay analyser according to the manufacturer’s instructions (Roche).

### Cell culture

Vero E6 (African green monkey, female, kidney*;* CRL-1586, ATCC, RRID : CVCL_0574) cells were grown in Dulbecco’s modified Eagle’s medium (DMEM, Gibco) which was supplemented with 10% heat-inactivated foetal calf serum (FCS), 100 units/ml penicillin, 100 µg/ml streptomycin, 2 mM L-glutamine, 1 mM sodium pyruvate, and 1× non-essential amino acids. HEK293T (human, female, kidney; ACC-635, DSMZ, RRID: CVCL_0063) cells were grown in DMEM with supplementation of 10% FCS, 100 units/ml penicillin, 100 µg/ml streptomycin, and 2 mM L-glutamine. All cells were grown at 37°C in a 5% CO_2_ humidified incubator. Cell lines were recently purchased from the indicated companies and used without further authentication. All cell lines were regularly tested for mycoplasma contamination and remained negative.

### Preparation of pseudotyped viral particles

Expression plasmids for vesicular stomatitis virus (VSV, serotype Indiana) glycoprotein (VSV-G) and SARS-CoV-2 spike variants Wuhan-Hu-1 D614G (B.1) (30), Delta (B.1.617.2) (31), and Omicron (B.1.1.529.1; BA.1) (9) (codon-optimized; with a C-terminal truncation for increased pseudovirus packaging) have been described elsewhere (32). Transfection of cells was carried out by Transit LT-1 (Mirus). Rhabdoviral pseudotype particles were prepared as previously described (33). A replication-deficient VSV vector in which the genetic information for VSV-G was replaced by genes encoding two reporter proteins enhanced green fluorescent protein and firefly luciferase (FLuc) and VSV∗ΔG-FLuc (34) (kindly provided by Gert Zimmer, Institute of Virology and Immunology, Mittelhäusern, Switzerland (34)) was used for pseudotyping. One day after transfection of HEK293T cells to express the viral glycoprotein, they were inoculated with VSV∗ΔG-FLuc and incubated for 1–2 h at 37°C. Then the inoculum was removed, cells were washed with PBS, and fresh medium was added. After 16–18 h, the supernatant was collected and centrifuged (2,000 × g, 10 min, room temperature) to clear cellular debris. Cell culture medium containing anti-VSV-G antibody (I1-hybridoma cells; ATCC no. CRL-2700) was then added to block residual VSV-G-containing particles. Samples were then aliquoted and stored at -80°C.

### Pseudovirus neutralization assay

For pseudovirus neutralization experiments, Vero E6 cells were seeded in 96-well plates 1 day prior (6,000 cells/well) in medium containing 2.5% FCS. Heat-inactivated (56°C, 30 min) sera were serially titrated (fourfold titration series with seven steps + buffer only control) in PBS, pseudovirus stocks added (1:1, v/v), and the mixtures incubated for 30 min at 37°C before being added to cells in duplicates (final on-cell dilution of sera: 20; 80; 320; 1,280; 5,120; 20,480; 81,920-fold). After an incubation period of 16–18 h, transduction efficiency was analysed. For this, the supernatant was removed, and cells were lysed by incubation with Cell Culture Lysis Reagent (Promega) at room temperature. Lysates were then transferred into white 96-well plates, and luciferase activity was measured using a commercially available substrate (Luciferase Assay System, Promega) and a plate luminometer (Orion II Microplate Luminometer, Berthold). For analysis of raw values (RLU/s), the background signal of an uninfected plate was subtracted and values normalized to pseudovirus treated with PBS only. Results are given as serum dilution resulting in 50% pseudovirus neutralization (PVNT50) on cells, calculated by non-linear regression ([Inhibitor] vs. normalized response – Variable slope) in GraphPad Prism Version 9.1.1.

### Determination of SARS-CoV-2 spike-specific CD4^+^ and CD8^+^ T cell responses by intracellular cytokine staining (ICS)

Cryopreserved PBMCs of study participants were thawed and rested overnight at 37°C with 1 µl/ml of DNase (DNase I recombinant, RNase-free (10,000 U) Roche), in RPMI medium supplemented to contain a final concentration of 10% FCS, 10 mM HEPES, 1× MEM non-essential amino acids (Corning Life Sciences/Media Tech Inc., Manassas, VA), 1 mM sodium pyruvate (Lonza, Walkersville, MD, USA), 1 mM penicillin/streptomycin, and 1× 2-mercaptoethanol (Gibco, Invitrogen, Carlsbad, CA, USA). Stimulation of PBMCs for detection of cytokine production by T cells was adapted from Kasturi et al. (2020) (35). Briefly, 1 × 10^6^ PBMCs were cultured in 200 μl final volume in a 96-well U bottom plate in the presence of 1 µg/ml anti-CD28 and anti-CD49d (BioLegend) under the following conditions: a) negative DMSO control, b) 2 μg/ml SARS-CoV-2 spike peptide pools (1-315 peptides from Wuhan-Hu-1, Delta (B.1.617.2), and Omicron (B.1.1.529.1; BA.1) SARS-CoV-2 spike, JPT Germany), c) 2 μg/ml of CEFX Ultra Super Stim peptide pool (176 peptide epitopes for a broad range of HLA subtypes of 18 different infectious agents including clostridium tetani, coxsackievirus B4, influenza A virus, haemophilus influenza, helicobacter pylori, human adenovirus 5, human herpesvirus 1/2, human herpesvirus 3, human herpesvirus 4, human herpesvirus 5, human herpesvirus 6, human papillomavirus, JC polyomavirus, measles virus, rubella virus, toxoplasma gondii, and vaccinia virus, JPT Germany) as SARS-CoV-2 vaccination-independent control of d) positive control phorbol 12-myristate 13-acetate (PMA) (50 ng/ml) and ionomycin (500 ng/ml). Cells were cultured for 2 h before adding 10 μg/ml brefeldin A (Sigma-Aldrich, St. Louis, MO) for an additional 5 h. Cells were then washed with PBS and prestained for dead cells (Live/Dead Fixable; Aqua from Thermo Fisher) and for the chemokine receptor 7 by APC/Cy7-anti-human CCR7 (clone G043H7) for 30 min at 37°C, 5% CO_2_. Cells were incubated with surface antibody cocktail (prepared in 1:1 of FACS buffer and brilliant staining buffer) for 30 min at room temperature with BV510-anti-human CD14 (clone M5E2), BV510-anti-human CD19 (clone HIB19), AF700 anti-human CD3 (clone OKT3), BV605 CD4 (clone OKT4), PerCP-Cy5.5 CD8 (clone RPA-T8), and PE/Fire 700-anti-human CD45RA (clone HI100) from BioLegend. Next, cells were fixed using Cytofix/Cytoperm buffer (BD Biosciences, CA) for 20 min at room temperature and then kept in FACS buffer at 4°C overnight. Perm/Wash (1×, BD Biosciences, CA) was used for cell permeabilization for 10 min at room temperature followed by intracellular staining for 30 min at room temperature with AF647 anti-human IFNγ (clone 4S.B3) and AF488 anti-human IL-2 (clone MQ1-17H12) from BioLegend, and PE/Cy7 anti-human TNFα (clone Mab11) from Thermo Fisher Scientific. Up to 100,000 live CD3^+^ T cells were acquired on an LSRFortessa flow cytometer (BD Biosciences), equipped with FACSDiva software. Analysis of the acquired data was performed using FlowJo software (version 10.7.1). The background was corrected by subtracting the signal of the DMSO control from the spike-treated cells.

### Statistical analysis

The SARS-CoV-2 convalescent individual was excluded in all statistical analyses. Non-parametric Spearman rank correlation was used to check for possible associations at single blood sample measurements. To include neutralizing antibody titers lower than the detection limit of 20, values were set to 10. Longitudinal antibody measurements were analysed by means of a mixed linear regression model including a random intercept to account for the repeated-measure structure of the underlying data. The mixed linear model approach enabled to simultaneously account for possible confounding due to participants’ age and for the presence of missing data (36). Therefore, no formal imputation of missing interim values was required. Comparison between variants and of T cell responsiveness was done by the Mann–Whitney-U test because of skewed distributions and with Wilcoxon signed-rank test for matched pairs. A two-sided alpha error of 5% was applied to analyses. All analyses were done by GraphPad Prism version 9.1.1 for Windows, GraphPad Software, San Diego, CA, USA, www.graphpad.com, R (version 4.0.1) and SAS (version 9.4).

## Results

A previously described cohort of 26 individuals, of whom one had a history of SARS-CoV-2 infection, received a primary vaccination of a heterologous ChAdOx1 nCoV-19 prime and BNT162b2 boost within an 8-week interval in early 2021 (25). We performed a follow-up study of this young cohort (23 participants, median age 29.5 years, **Table 1**) for a duration of 7 months after primary vaccination and analysed humoral and cellular immunity over time, as well as reactogenicity and immune responses after a BNT162b2 ‘booster’ vaccination 6.5 months later (18 participants).

**Figure 1:**
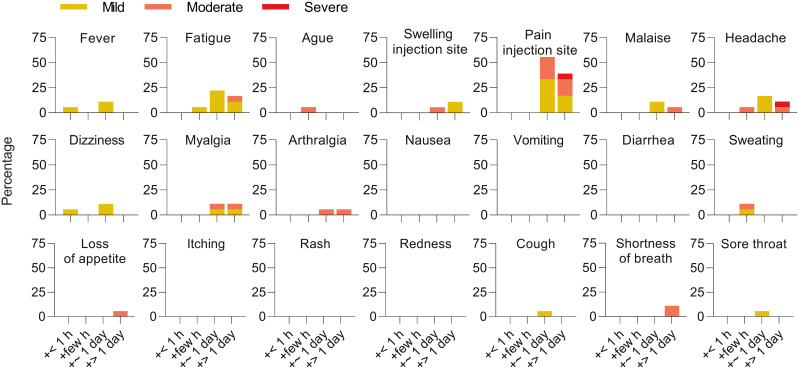
Reactogenicity of a ‘booster’ after heterologous primary vaccination. Solicited adverse reactions following BNT162b2 ‘booster’ vaccination. Percentages of n = 18 participants with individual symptoms following vaccination are shown. Severity is graded on a scale of 1–2 (for some symptoms) or 1–3 (for most), as adapted from the Common Terminology Criteria for Adverse Events (US Department of Health and Human Services, Version 4.03).

The ‘booster’ vaccination was well tolerated and associated with a lower overall reactogenicity compared to the initial two heterologous doses (25). The major solicited adverse reactions were pain at the injection site (94%, 17/18 participants), fatigue (44.4%, 8/18), and headache (33.3%, 6/18). No serious adverse events were observed (**Figure 1**).

**Figure 2:**
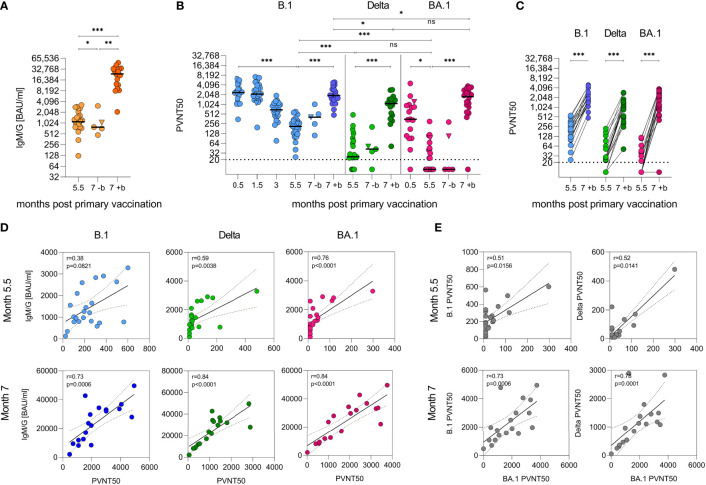
Humoral immunity against SARS-CoV-2 after heterologous vaccination followed by a ‘booster’ vaccination. **(A)** Quantification of cumulative anti-SARS-CoV-2 spike IgG and IgM responses as binding antibody units per ml (BAU/ml) by immunoassay with (+b) or without (-b) ‘booster’ after 6.5 months. **(B)** VSV-based B.1, Delta, and Omicron (BA.1) SARS-CoV-2 spike pseudovirus neutralization assay. Titers expressed as serum dilution resulting in 50% pseudovirus neutralization (PVNT50) were obtained from three experiments in duplicate infections. Triangle indicates SARS-CoV-2 convalescent individual, who was excluded from all statistical analyses. Dashed horizontal lines indicate lower limit of detection. Samples were obtained from n = 23 participants. Booster samples were taken 2 weeks after vaccination. Longitudinal antibody measurements were analysed by means of a mixed linear regression model. **(C)** Data from **(B)** illustrated as paired values pre and post ‘booster’. **(D)** Spearman correlation of IgG/IgM and neutralizing titers and **(E)** between neutralizing titers, two-tailed p values, dashed lines indicate 95% confidence interval. ***p < 0.001, **p < 0.01, *p < 0.05, ns, not significant..

As described previously (25), 2 weeks after primary vaccination, the cohort showed median cumulative anti-SARS-CoV-2-spike IgM and IgG (IgM/G) titers of 8,815 (1,206–19,046) BAU/ml, which decreased to 2,039 (235–5,926) BAU/ml over the course of 3 months. In the (slightly smaller) follow-up cohort, they further declined to 1,120 (125–3,287) BAU/ml after 5.5 months, corresponding to an eightfold decrease (**Figure 2A**). After 6.5 months, 18 of the participants (median 1,243 BAU/ml) received a BNT162b2 ‘booster’. Two weeks later, the median IgM/G titers had increased by 21-fold to 25,775 BAU/ml (2,092–49,627; p < 0.001, mixed model) in boosted individuals, while further decreasing to 753 (474–3,076, p = 0.0186, mixed model) BAU/ml in non-boosted participants (**Figure 2A**). This corresponds to a 34-fold higher median titre in the boosted versus non-boosted group at the 7-month time point (p = 0.0033, mixed model) and exceeds the initial titers determined after the primary vaccination by ~2-fold [median 11,339 BAU/ml (25)].

Using vesicular stomatitis virus (VSV)-based pseudoviruses (PVs) carrying the SARS-CoV-2 spike protein, we analysed the neutralizing activity of the sera. Two weeks after the primary vaccination, median 50% pseudovirus neutralization (PVNT50) titers against PV carrying the SARS-CoV-2 Wuhan-Hu-1 D614G (B.1) spike protein were 2,418 (350–6,383). PVNT50 titers remained stable for 1.5 months but decreased 12-fold to 204 (24–601) over the course of 5.5 months (**Figure 2B**, p < 0.0001, mixed model). In comparison, titers against the Delta VOC after 5.5 months were eightfold lower with median titers of 24 (<20–481) (**Figures 2B**, **S1**, p < 0.0001, mixed model). In contrast to studies on homologous vaccinations, but in line with other studies on sera from heterologously vaccinated individuals (9, 15, 37), median neutralization titers of 345 (<20–4541) were already detected against the Omicron BA.1 VOC 2 weeks after primary vaccination in 15/16 (94%) participants. These titers decayed to <20 (<20–299) after 5.5 months, with 12/22 (55%) participants showing no detectable neutralizing activity at all. This corresponds to a 7–20-fold immune evasion compared to B.1 (**Figures 2B**, **S1**, p < 0.0001, mixed model). After the ‘booster’, titers against the B.1 variant increased ninefold to 1,929 (474–4,942), 45-fold to 1,094 (51–2,895) for Delta, and >88-fold to 1,768 (<20–3,760) against BA.1 (**Figures 2B, C**, **S1**, p < 0.0001, mixed model). Strikingly, the neutralizing titers 2 weeks after the ‘booster’ against Delta and BA.1 were similar and only slightly lower than for B.1 (**Figures 2B, C**, **S1**, p = 0.9608, p = 0.0198, p = 0.0211, mixed model). At 5.5 months after primary vaccination, the neutralizing activity correlated weakly with IgM/G titers; however, after the ‘booster’ (7 months after primary vaccination), the correlation was highly significant for all variants, indicating that induction of high titers is associated with potent neutralization (**Figure 2D**, r ≥ 0.73, Spearman). Notably, the neutralizing titers obtained for the three variants correlated only weakly before but became strongly significant after the ‘booster’ immunization (**Figure 2E**, r ≥ 0.73, Spearman). These results indicate that the ‘booster’ induces broadly neutralizing antibodies that are even effective against the highly divergent Omicron BA.1 variant. Results were not confounded by participant age or sex.

**Figure 3:**
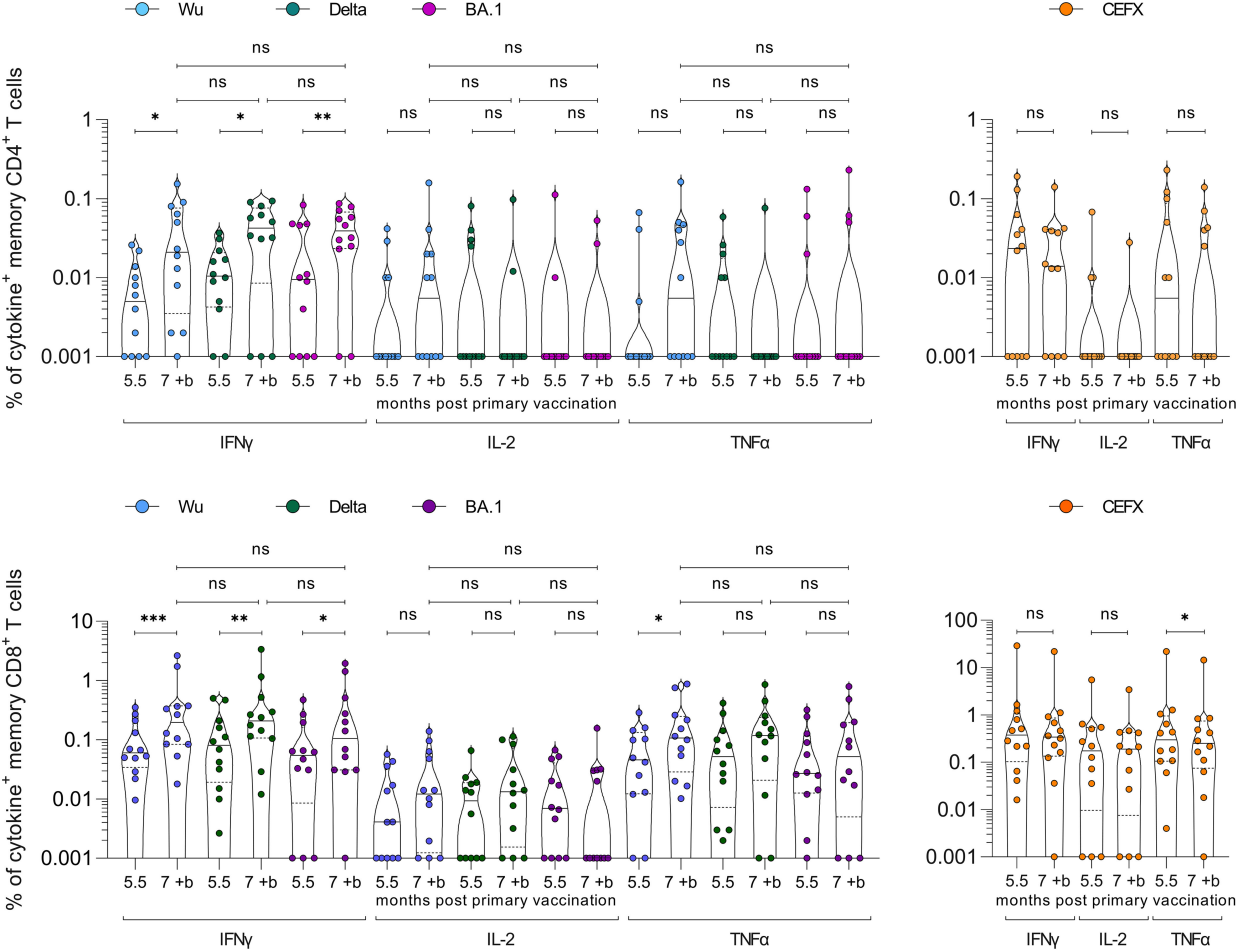
SARS-CoV-2 spike-specific CD4^+^ and CD8^+^ memory T cell responses after heterologous vaccination followed by a ‘booster’ vaccination. PBMCs isolated from samples of n = 12 study participants were obtained 5.5 months after the heterologous primary vaccination, and 2 weeks after the BNT162b2 ‘booster’ (7 months post primary vaccination). PBMCs were stimulated with SARS-CoV-2 Wuhan-Hu-1 (Wu), Delta, or Omicron (BA.1) spike peptide-pool (left panels) or control pools of different infectious agents (CEFX, right panels) and cytokine production determined by flow cytometry. CD4^+^ (upper panel) and CD8^+^ (lower panel) memory T cells were gated and analysed for IFNγ, IL-2, and TNFα cytokine production. Cytokine^+^ T cells were background-corrected for unstimulated cells (**Figures S2**, **S3**), and zero values set to 0.001%. Wilcoxon matched-pair signed-rank test compares cytokine-positive cells before and after the ‘booster’. Mann–Whitney-U test compares cytokine-positive cells post ‘booster’ between variants. ***p < 0.001, **p < 0.01, *p < 0.05, ns, not significant.

To evaluate cellular immunity, we isolated peripheral blood mononuclear cells (PBMCs) from blood samples provided by 12 participants 5.5 months after the primary vaccination, as well as samples 2 weeks after the ‘booster’. Cells were exposed to pools of 315 peptides spanning the spike sequences of SARS-CoV-2 Wuhan-Hu-1 (Wu), Delta (B.1.617.2), or Omicron (B.1.1.529.1; BA.1) and analysed for intracellular cytokines IFNγ, IL-2, and TNFα. Increased cytokine production upon peptide stimulation was evaluated to determine responsive and spike-specific CD4^+^ and CD8^+^ memory T cells (**Figures S2, S3**). At 5.5 months after primary vaccination, only five of the 12 donors showed remaining CD4^+^ memory T cells responding to either spike peptide stimulation by IL-2 or TNFα production, respectively (**Figure 3**). In contrast, most participants (10 of 12) showed remaining memory CD4^+^ T cells responding by IFNγ production (median 0.005%–0.011% reactive cells). Notably, the magnitude of responses to SARS-CoV-2 BA.1 spike did not differ from Wu or Delta (**Figure 3**, p > 0.05, Mann–Whitney-U), indicating efficient cross-reactivity. After the ‘booster’, spike-specific IFNγ CD4^+^ memory T cell responses and the fraction of reactive cells further increased to 11 of 12 participants and the median ranged from 0.02% to 0.04% responsive cells for the spike peptide variants (p = 0.0273, p = 0.0137, p = 0.0098; Wilcoxon signed-rank). However, CD4^+^ T cells responding by IL-2 or TNFα secretion were not affected by the ‘booster’. CD8^+^ memory T cells showed a longer durability and typically remained reactive over the course of 5.5 months, with 8 of 12 participants responding to Wu, Delta, or BA.1 spike peptide challenge by IL-2 (0.004%–0.009%), all by TNFα (0.027%–0.052%) and IFNγ production (0.055%–0.081%) (**Figure 3**). Again, the ‘booster’ significantly enhanced IFNγ responses for all variants (0.105%–0.208%) (**Figure 3**, p = 0.0005, p = 0.0049, p = 0.0137; Wilcoxon signed-rank). IL-2 and TNFα responses also showed an increase, but not significant. Stimulation with a pool of 176 peptide epitopes from 18 infectious agents (CEFX) confirmed that the ‘booster’ did not unspecifically affect T cell responses. Of note, 11 of 12 participants developed CD4^+^ and all participants CD8^+^ T cell memory against BA.1. Altogether, 5.5 months after heterologous vaccination participants showed stable CD8^+^ memory T cell levels but a decreased humoral and CD4^+^ memory T cell immunity. A BNT162b2 ‘booster’, however, reactivated and enhanced T cell immunity and induced potent antibody responses also against the Omicron BA.1 VOC.

## Discussion

Heterologous primary ChAdOx1 nCoV-19 prime, BNT162b2 boost vaccination induces potent immune responses against SARS-CoV-2 (25–28) resulting in effective protection from COVID-19 (24). Data about long-term immunity and protection conferred by this vaccination regimen, as well as reaction toward a ‘booster’ vaccination and its efficacy toward the Omicron VOC, are, however, scarce, in particular for younger individuals (24). T cells generally show broad cross-reactivity against SARS-CoV-2 variants (25, 38) including Omicron (39–41), which is expected, because the majority of mutations in the Omicron spike are not located in known T cell epitopes (42, 43) and because the large HLA allele diversity on population level makes T cell evasion unlikely (44). In contrast, BA.1 showed neutralization-evading properties (9–14, 20, 21) which consequently results in loss of protection from symptomatic infection (45–47). Thus, ‘booster’ vaccinations are performed aiming for enhanced immune protection especially from Omicron (9–14, 16, 19–21). ‘Booster’ vaccinations after homologous BNT162b2 or ChAdOx1 nCoV-19 vaccination regimen have been described as safe (22) and shown to restore protection from the Delta VOC (17, 18) and to reduce Omicron breakthrough infections and the secondary attack rate (48).

We here show for a young cohort with a median age of 29.5 years that heterologous primary vaccination already resulted in antibody titers with moderate Omicron BA.1-neutralizing activity, which declined over the course of 5.5 months to levels hardly neutralizing this VOC. In line, spike-specific CD4^+^ memory T cells showed remaining but limited reactivity 5.5 months after heterologous vaccination. However, CD8^+^ memory T cells remained responsive and also reacted to BA.1 spike epitopes. This is in line with the observation that individuals that received homologous primary vaccination remain partly protected from hospitalization upon Omicron infection (49–51) but also with the fact that Omicron shows increased breakthrough infections (52). The ‘booster’ resulted in lower reactogenicity than determined in the first two vaccinations (25) and elicited both high antibody titers and enhanced memory T cell responses against the tested SARS-CoV-2 variants including BA.1. Strikingly, the induced neutralizing antibodies were as potent against BA.1 as against Delta and almost as potent as against B.1. This is in contrast to earlier studies focusing on homologous short-interval primary vaccinations that also found ‘boosters’ to induce BA.1-neutralizing titers but where this variant still shows some degree of evasion (9–14, 20, 21). This highlights the major benefits of a third dose regimen (50, 51, 53) especially after heterologous primary vaccination in young adults to protective humoral and cellular immunity against SARS-CoV-2 variants.

In this cohort, the BNT162b2 ‘booster’ induced titers that were neutralizing BA.1 almost as potently as B.1. In light of the immune evasive properties of Omicron and the results from studies on homologous vaccinations, this finding is somewhat surprising. As potential confounding factors, unnoticed SARS-CoV-2 infection of the participants was excluded by nucleocapsid antibody detection and adequate sensitivity of the used neutralization assay has been validated previously (54). Therefore, this exceptionally efficient neutralization of BA.1 is most likely due to the extremely high antibody levels after the ‘booster’ as well as the long-term germinal center reaction, ongoing affinity maturation after vaccination (55), and reactivation of memory B cells (56). The Delta VOC was also potently neutralized after the ‘booster’, resulting in titers that were lower than against B.1 but similar to BA.1, indicating immune evasion by these VOCs but at the same time a broad cross-reactivity of ‘booster’-induced antibodies. These remarkable potent and broadly active antibodies might be a result of the heterologous vaccination where DNA and mRNA vaccines encoding non- and pre-fusion-stabilized spike protein variants are mixed (57). Another explanation for the strikingly effective neutralization of the Omicron BA.1 VOC might be the relatively young age and associated potent immunity (58–61) of the here analysed cohort with median age of 29.5 years. Also timing might play a role, as the intervals in the primary vaccination and between the ‘booster’ influence humoral as well as cellular responses (62, 63). Studies on homologous primary BNT162b2 vaccinations with a 3-week interval detected neutralizing titers of 306–604 against B.1 but only undetectable-13 against BA.1 (9–11, 20, 21). In our study, we already detected a titre of 345 after primary vaccination, and similar results have been obtained in other studies looking at BA.1 neutralization of sera from heterologously ChAdOx1-BNT162b2-vaccinated individuals with an interval of 8–12 weeks (9, 15, 37). This is in line with the finding that longer intervals in heterologous or homologous primary vaccinations result in higher neutralization capacities of SARS-CoV-2 VOCs (21, 28, 62, 64–66). This might be attributed to ongoing antibody maturation before primary boost (55, 56, 67) as has also been observed for vaccination against influenza virus (68). Thus, the typically longer interval within heterologous primary vaccination might result in affinity maturation already before the second vaccine dose and explain the strikingly potent cross-neutralization of BA.1 after the ‘booster’.

After the ‘booster’, memory CD4^+^ T cells became strongly reactive toward SARS-CoV-2 spike peptides of all variants. Also the reactivity of the residual CD8^+^ T cells was further enhanced by the ‘booster’ dose, which agrees with data after homologous primary vaccinations showing that a third dose enhances preexisting cellular responses (69, 70). The finding that SARS-CoV-2-specific T cells are generally reactive to spike peptides derived from the Omicron VOC is supported by recent data (19, 40, 70) and suggests that the ‘booster’ might also enhance protection of Omicron-infected individuals from severe disease (71).

The Omicron VOC seems to form a new antigenic cluster (72–74), of which the BA.2 variant has rapidly expanded, now followed by BA.5. First results indicate that neutralization capacity is similar between BA.1 and BA.2 (75, 76) and lower against BA.5 (77, 78); however, the general cross-reactivity of T cells suggests that the ‘booster’ is likely also effective against these variants. Therefore, it will now be of importance to elucidate the longevity of humoral and cellular immunity after the ‘booster’ against circulating variants and most importantly its durable effectiveness in preventing infection and disease (79). In addition, fourth vaccine doses (80) or an adaptation of the vaccines to BA.1 (81) need yet to be proven useful to provide effective protection from these old and new variants. Yet unvaccinated individuals might benefit from an updated vaccine that establishes a high degree of protection against Omicron already after two doses. Altogether, our results suggest that a ‘booster’ 6 months after initial heterologous vaccination of young adults induces good humoral and cellular protection against SARS-CoV-2 Omicron, even though the antigen of immunization is the ancestral Wuhan-Hu-1 spike. Thus, a ‘booster’ following the heterologous vaccination is highly warranted especially in the light of the immune evading Omicron variants.

## Data availability statement

The raw data supporting the conclusions of this article will be made available by the authors, without undue reservation.

## Ethics statement

The studies involving human participants were reviewed and approved by the ethics committee of Ulm University. The patients/participants provided their written informed consent to participate in this study.

## Author contributions

Conceptualization, JMü, JM, AS, MZ, RG. Funding acquisition, JMü, JM, FK, SP. Investigation, AS, MZ, RG, DK, SE-E, DA, CC, SL, PM, TW, AB, JK, JMü. Essential resources, BM, MH, SP, FK, JM, JMü. Writing, JMü, AS, MZ, RG. Review and editing, all authors. AS, MZ, RG contributed equally. AS, MZ, RG and JMü verified the underlying data. All authors read and approved the final version of the manuscript.

## Funding

This project has received funding from the European Union’s Horizon 2020 research and innovation programme under grant agreement no. 101003555 (Fight-nCoV) to JM, the German Research Foundation (CRC 1279) to FK and JM, MU 4485/1-1 to JMü, the BMBF to FK (Restrict SARS-CoV-2), and the Robert Koch Institute (RKI). Additional funding was provided to SP from BMBF (01KI1723D, 01KI20328A, 01KI20396, 01KX2021) and the county of Lower Saxony (14-76103-184, MWK HZI COVID-19) and to JM by the DFG (KL 2544/8-1, FR 2974/3-1).

## Acknowledgments

We thank all participants for regular blood donations. We thank Nicola Schrott, Regina Burger, Jana Romana Fischer, and Birgit Ott for skilful laboratory assistance. We thank Sarah Warth and Simona Ursu of the flow cytometry core facility for support with flow cytometric analysis. R.G., A.S., C.C, and T.W. are part of the International Graduate School in Molecular Medicine Ulm.

## Conflict of interest

The authors declare that the research was conducted in the absence of any commercial or financial relationships that could be construed as a potential conflict of interest.

## Publisher’s note

All claims expressed in this article are solely those of the authors and do not necessarily represent those of their affiliated organizations, or those of the publisher, the editors and the reviewers. Any product that may be evaluated in this article, or claim that may be made by its manufacturer, is not guaranteed or endorsed by the publisher.
