# Supplementary material for: BNT162b2 booster after heterologous prime-boost vaccination induces potent neutralizing antibodies and T cell reactivity against SARS-CoV-2 Omicron BA.1 in young adults
Source: Front Immunol. 2022 Jul 25;13:882918. doi: 10.3389/fimmu.2022.882918 (PMC9357986; doi:10.3389/fimmu.2022.882918)
Supplement: Supplementary file 1 [file DataSheet_1.docx]

Supplementary Material

# Supplementary Figures

**
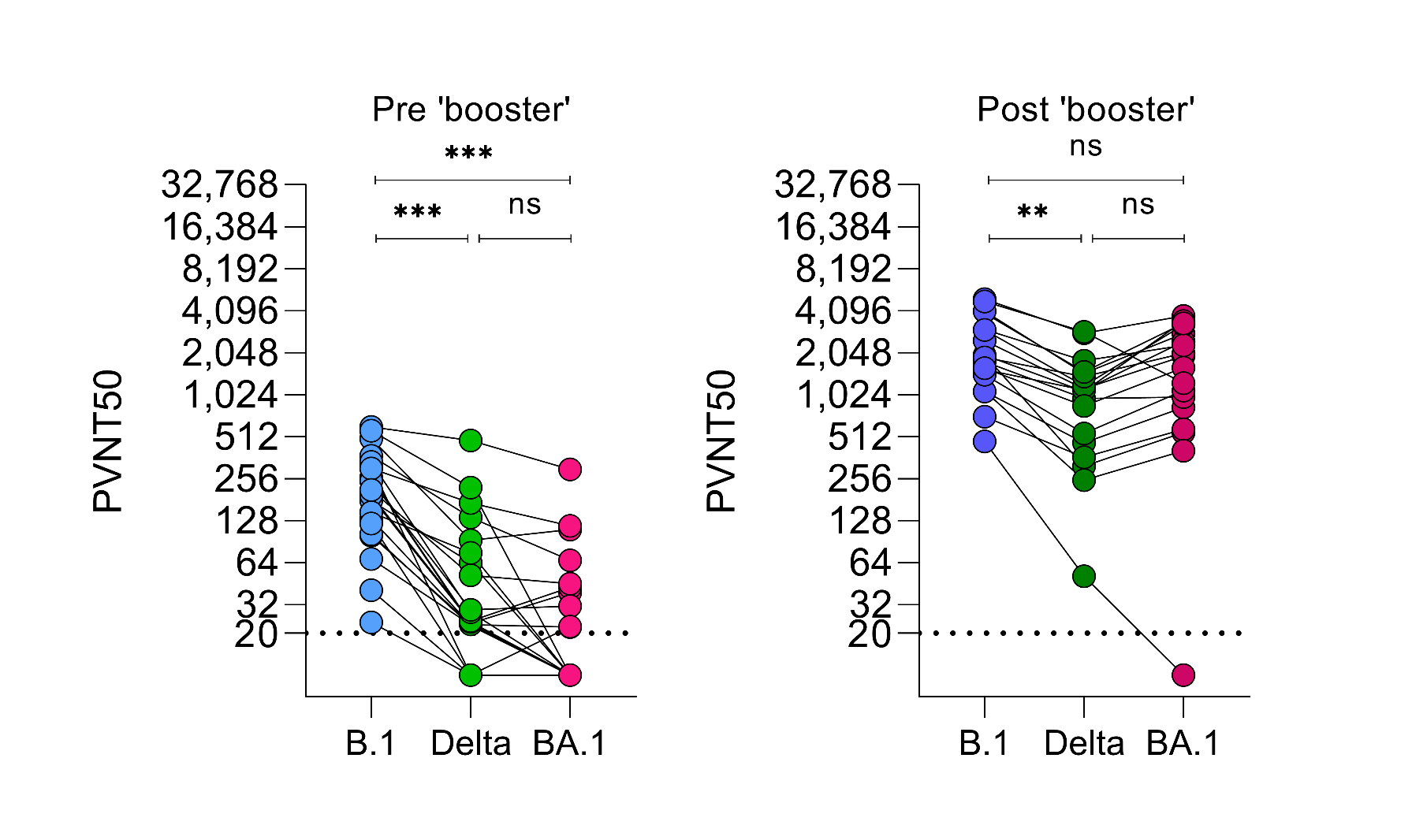
**

**Supplementary Figure 1. Individual neutralization of VOCs.** Data from Figure 2, pre ‘booster’ (5.5 months post primary vaccination) and post ‘booster’ (7 months post primary vaccination) illustrated as paired neutralization titers against B.1, Delta, and Omicron (BA.1). Mann-Whitney-U test *** p < 0.001, ** p < 0.01, ns not significant


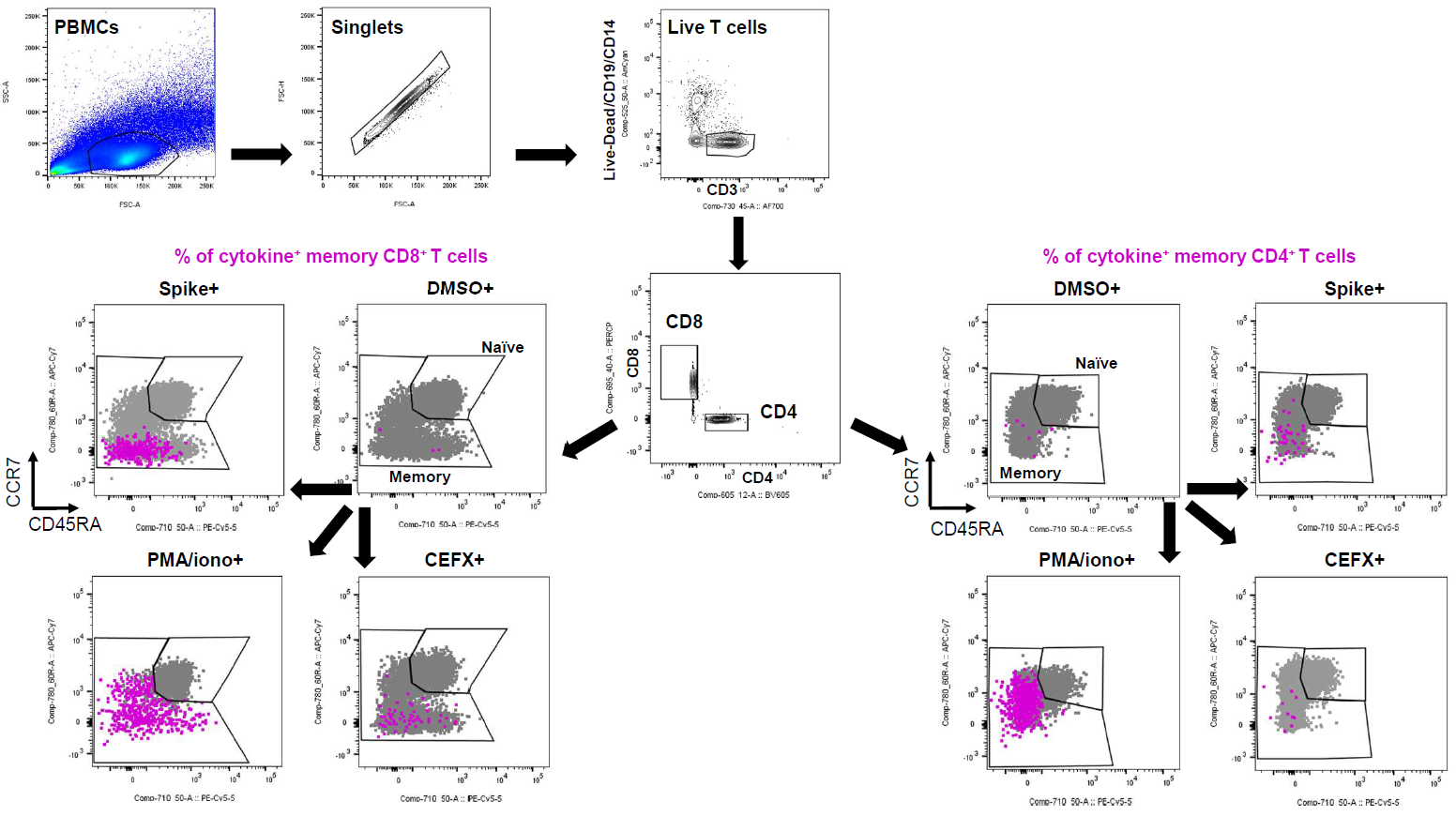


**Supplementary Figure 2. Gating strategy for analysis of memory T cell reactivity.** Unstimulated (DMSO) and stimulated (SARS-CoV-2 spike peptide, CEFX, PMA/ionomycin) PBMCs were initially gated based on light scatter (SSC-A versus FSC-A) and for singlets (FSC-H versus FSC-A). Dead cells, monocytes, and B cells were excluded using a dump channel and by gating on CD3^+^ T cells. Total CD8^+^ and CD4^+^ cells were then selected, and individual cytokine gating was performed in the whole memory/non-naïve CD8^+^ and CD4^+^ T cell subsets as defined by expression of CD45RA and CCR7.


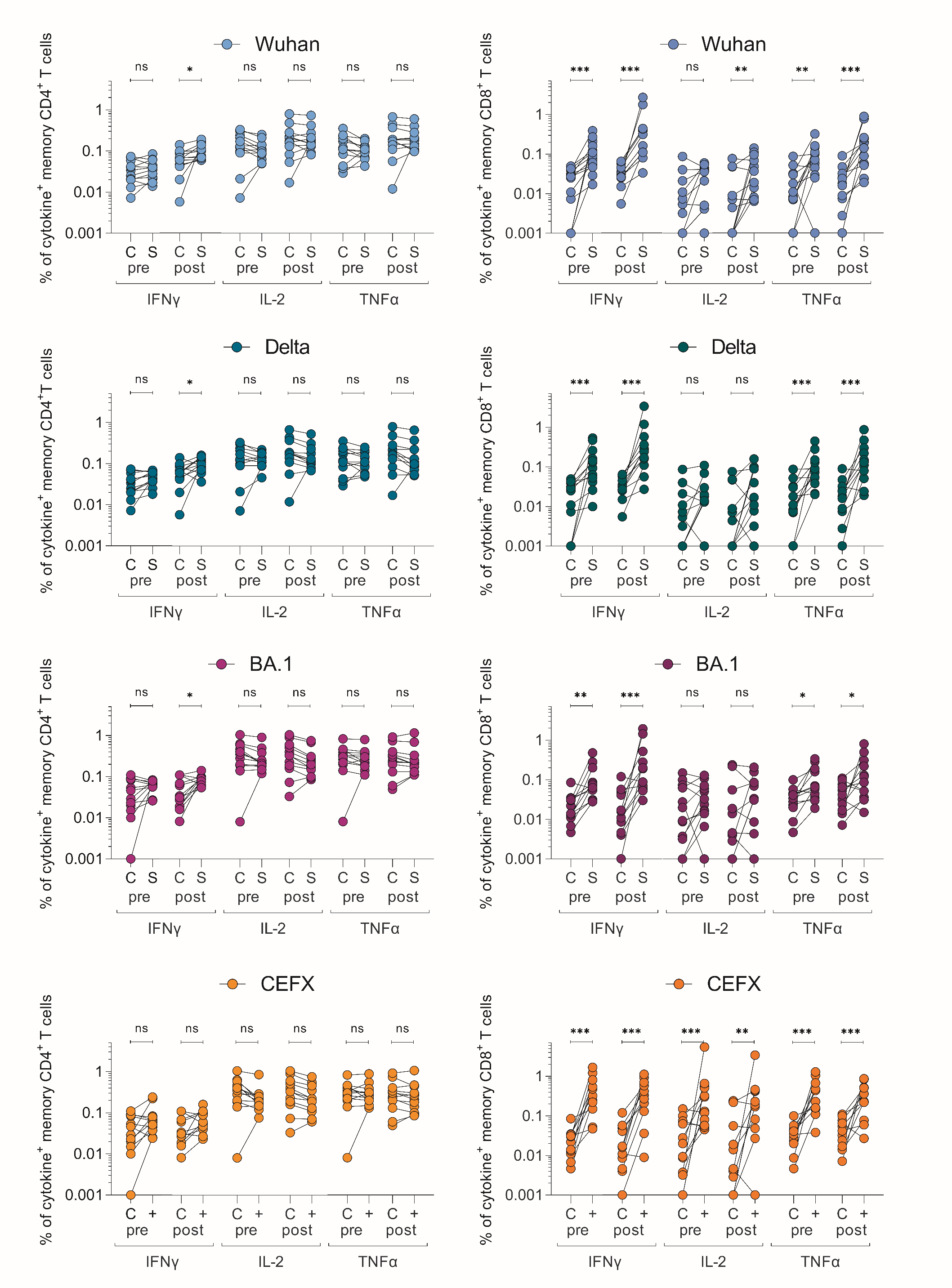


**Supplementary Figure 3.** **SARS-CoV-2 spike-specific CD4^+^ and CD8^+^ memory T cell responses before and after the ‘booster’.** PBMCs 5.5 months after primary vaccination (pre) and two weeks after the ‘booster’ (7 months after primary vaccination, post) of n = 12 study participants were stimulated with SARS-CoV-2 Wuhan-Hu-1 (Wu, blue), Delta (B.1.617.2, green), Omicron (B.1.1.529.1; BA.1, purple) spike peptides (S) or a DMSO control (C). As positive control (+), pools of epitopes from different infectious agents (CEFX, orange) were used for stimulation. CD4^+^ (upper panel) CD8^+^ memory T cells (lower panel) and IFNγ, IL-2, or TNFα cytokine production were determined by flow cytometry. Mann-Whitney-U test compares T cell responses upon stimulation by control versus SARS-CoV-2 spike-peptide or CEFX treatment. *** p < 0.001, ** p < 0.01, * p < 0.05; ns = not significant.
